# Sainfoin in the Dams’ Diet as a Source of Proanthocyanidins: Effect on the Growth, Carcass and Meat Quality of Their Suckling Lambs

**DOI:** 10.3390/ani12040408

**Published:** 2022-02-09

**Authors:** Clàudia Baila, Sandra Lobón, Mireia Blanco, Isabel Casasús, Guillermo Ripoll, Margalida Joy

**Affiliations:** Departamento de Ciencia Animal, Centro de Investigación y Tecnología Agroalimentaria de Aragón (CITA), Instituto Agroalimentario de Aragón–IA2 (CITA-Universidad de Zaragoza), Avda. Montañana 930, 50059 Zaragoza, Spain; cbaila@cita-aragon.es (C.B.); slobon@cita-aragon.es (S.L.); mblanco@cita-aragon.es (M.B.); icasasus@cita-aragon.es (I.C.); gripoll@cita-aragon.es (G.R.)

**Keywords:** *Onobrychis viciifolia*, condensed tannins, performance, plasma metabolites, meat color

## Abstract

**Simple Summary:**

Several studies point out that the use of local forage legumes, such as sainfoin, can be appropriate for feeding sheep autochthonous breeds, with additional benefits also for the soil. Besides, sainfoin has a medium content of proanthocyanidins (PAC), also known as condensed tannins, the effects of which have been studied in fattening lambs but seldom on suckling lambs. The aim of the study was to evaluate the effect of PAC of sainfoin fed to dams on the productive traits, weight of the digestive organs, and on carcass and meat quality of their suckling lambs. The inclusion of PAC from sainfoin in the dam diet did not produce detrimental changes on the growth and carcass and meat characteristics of their suckling lambs. Therefore, sainfoin can be fed to ewes during lactation to produce suckling lambs, achieving good performances and meat quality.

**Abstract:**

Sainfoin (*Onobrychis viciifolia*) is a forage legume with a medium content of proanthocyanidins (PAC), which may affect animal performance and product quality. The objective of the present study was to assess the effect of PAC from sainfoin fed to dams, using polyethylene glycol (PEG) as a blocking agent, on the performance and carcass and meat quality of their suckling male lambs. After lambing, twenty lactating dams were fed fresh sainfoin *ad libitum* plus 200 g per day of barley; ten were orally dosed with water (Sainfoin), and ten were dosed orally with a water dilution of 100 g PEG (Sainfoin + PEG). Their lambs (4.1 ± 0.64 kg at birth) suckled *ad libitum* until they reached the target slaughter weight of 10–12 kg. The presence of PAC in the dams’ diet did not affect the growth, blood metabolites and carcass weight and fatness of the suckling lambs but decreased the lightness of caudal fat (*p* < 0.05) and increased the weight of the digestive compartments (*p* < 0.05). Regarding the meat characteristics, PAC only decreased polyphenols content (*p* < 0.05). In conclusion, the presence of PAC in the dams’ diet had not significant effects on the performance and product quality of their suckling lambs.

## 1. Introduction

Nowadays, there is increasing social pressure for livestock production systems to minimize their negative environmental impacts, and to reduce the inclusion of feed components that compete for land use with human food crops [1]. In the last decades, the European Union has encouraged the use of local legumes for animal feeding in order to reduce the dependency on soybean meal, and to benefit from their positive environmental effects [2,3]. Among legume forages, sainfoin (*Onobrychis viciifolia*) has proven to be an excellent forage to be fed during lactation in ewes [4]. Furthermore, consumers are increasingly aware of the importance of food quality on human health, which has increased the demand for products obtained from forage-fed animals as they are considered healthier than those obtained from concentrate-fed diets [4,5], as well as more respectful with animal welfare.

In the Mediterranean area, the traditional production of suckling lambs —slaughtered at 10–12 kg body weight (BW)—is based on a system in which dams are fed diets mainly composed by straw, cereals and byproducts, and lambs are fed exclusively on their dams’ milk. In this framework, the inclusion of high proportions of fresh forage in the diet of the ewes could be an interesting alternative. In fact, grazing sainfoin during lactation improved the meat quality of light lambs even after a finishing period on concentrates, when compared to lambs reared with dams grazing alfalfa [6]. This could be due to a possible synergy between dietary proanthocyanidins (PAC) present in sainfoin and other antioxidant components in the muscle [7] or the milk [8].

Previous research concerning the effect of PAC on lamb performance is not conclusive, as some studies reported that weight increased [9,10], decreased [5] or did not change [11]. Regarding meat quality, there is also no consensus about the relation between PAC in lamb diets and the color parameters and heme pigments contents [12,13]. On the other hand, improvements in the antioxidant capacity of tissues due to the action of PAC have been observed [14,15]. Therefore, this great variability of results may depend on molecular weight, structure and degree of polymerization of PAC, as well as on the type of diet and animal studied [16].

Most of the studies regarding the inclusion of PAC have been carried out in fattening lambs with limited research on suckling lambs, the diet of which is based almost exclusively in milk. Therefore, to study the effect of PAC on the meat of suckling lambs, the source of PAC has to be included in their dam’s diet, and results cannot be extrapolated from those obtained in fattening lambs. Hence, the aim was to evaluate the effect of PAC of fresh sainfoin fed to dams on productive parameters, weight of the digestive organs, and carcass and meat quality of suckling lambs.

## 2. Materials and Methods

### 2.1. Experimental Site

The experimental procedures (CEEA, 2017–07), which were in compliance with the guidelines of the Directive 2010/63/EU of the European Parliament and of the Council of 22 September on the protection of animals used for experimental purposes, were approved by The Animal Ethics Committee of the Centro de Investigación y Tecnología Agroalimentaria de Aragón (CITA).

### 2.2. Animal Management and Experimental Design

The experiment was conducted in the facilities of CITA in Zaragoza, Spain —41°3′ N, 0°47′ W and 216 m above sea level— in spring 2019, during 28 days. All the methodology carried out during the experiment has been explained in detail in a previous study [17]. Briefly, after lambing twenty multiparous Rasa Aragonesa ewes with their male lambs were assigned into two homogeneous groups according to ewe body weight (BW; 61 ± 6.2 kg), body condition score (BCS; 3.3 ± 0.57), lambing date (April 6 ± 0.1 d) and lamb body weight at birth (4.1 ± 0.64 kg). All dams were fed fresh sainfoin (*Onobrychis viciifolia* cv Reznos) —dry matter (DM): 213 g/kg; crude protein (CP): 116 g/kg DM; neutral detergent fiber (NDF): 369 g/kg DM; acid detergent fiber (ADF): 264 g/kg DM; total PAC: 38.8 g eq. PAC sainfoin/kg DM—, water and mineral blocks ad libitum and 200 g/head/day of barley —DM: 912 g/kg; NDF: 250 g/kg DM; ADF: 87 g/kg DM; CP: 95 g/kg DM— distributed in two meals. Before each meal, ten ewes were drenched with 100 mL of water (Sainfoin), whereas ten ewes were orally dosed with 100 mL of polyethylene glycol (PEG) solution (50 g of PEG 4000/100 mL; Sainfoin + PEG to inactivate the effects of PAC. Each pair of dam-lamb was placed in an individual pen (2.2 m^2^). Lambs exclusively suckled their dams ad libitum until they reached the target slaughter weight of 10–12 kg BW.

The detailed chemical composition of feedstuffs and milk has been reported in a previous study [17]. The dry matter intake (1879.5 ± 281.3 g DM/d) and the milk yield and chemical composition was similar between groups —milk yield: 1.25 L/d; crude fat: 6.54%, CP: 5.02%, lactose: 5.28%—, except for the polyphenols (42.3 vs. 51.8 µg eq. [gallic acid]/g fresh sample, for Sainfoin and Sainfoin + PEG, respectively) and urea (275 vs. 338 mg/L, for Sainfoin and Sainfoin + PEG, respectively).

### 2.3. Measurements and Sampling Procedures

Lambs were weighed weekly at 8:00 h with an electronic scale (0.1 kg precision) to calculate the average daily gain (ADG). Blood samples were obtained the day of slaughter, from the jugular vein into heparin tubes (Vaccuette, Madrid, Spain). Samples were immediately centrifuged—3000 × *g* for 15 min at 4 °C—and stored at −20 °C until the metabolites analyses were performed.

When lambs reached the target weight of 10–12 kg BW, they were stunned by a captive bolt pistol and exsanguinated in the experimental abattoir of the Research Centre, using standard commercial procedures and according to Council Regulation (EC) Nº 1099/2009. The contents of the digestive tract corresponding to the sections of reticulum-rumen, omasum-abomasum and duodenum-jejunum were extracted and weighed. Then, the empty digestive compartments were weighed. Hot carcass weight (HCW) was recorded without head and offal. After 24 h chilling at 4 °C in total darkness, the cold carcass weight (CCW) was obtained. The dressing percentage was calculated as:

(1) $$\frac{\mathrm{HCW}}{\mathrm{slaughter}\;\mathrm{weight}}\times 100$$

and the carcass shrinkage was calculated as:

(2) $$\left(\frac{\mathrm{HCW}-\mathrm{CCW}}{\mathrm{HCW}}\right)\times 100$$

The fatness degree of the carcasses was determined following the Community Scale for Classification of Carcasses of Ovine Animals and of Light Lambs [18] and scored from 1 (1−, very low) to 4 (4+, very high) following the scale of 1 (low), 2 (slight), 3 (average), and 4 (high). Caudal subcutaneous fat color was measured on tail root using a Minolta CM-2006 d spectrophotometer (Konica Minolta Holdings, Inc., Osaka, Japan), registering lightness (L*), redness (a*), and yellowness (b*), which were used to calculate hue angle (h*_ab_*), and chroma (C*_ab_). The absolute value of the summation of the translated spectrum (SUM) was calculated as:

(3) $$SUM=\left[\left(\frac{TR_{450}}{2}\right)+TR_{460}+TR_{470}+TR_{480}+TR_{490}+TR_{500}+\left(\frac{TR_{510}}{2}\right)\right]\cdot 10$$

where TR_i_ was the reflectance value at i nm. An extensive explanation of the baselines of the method is exposed in Prache and Theriez [19].

After that, the carcass was carefully split longitudinally into the two half carcasses and the *longissimus thoracis et lumborum* (*LTL*) muscles of both sides were collected. Perirenal fat deposit was extracted and weighed.

### 2.4. Meat Quality

The *LTL* muscles from 4th–6th lumbar vertebrae of the left side were used to measure the pH with a pH-meter equipped with a Crison 507 penetrating electrode (Crison Instruments, S.A., Barcelona, Spain) and to estimate the chemical composition by NIRs (FoodScan^TM^2, Foss Analytics, Hilleroed, Denmark). From 6th to 13th thoracic *vertebrae* from both sides were sliced into 2.5 cm-hick samples, the left ones were assigned to days 0, 2 and 7 of display and the right ones for days of display 5 and 9. The slices were placed in trays, wrapped with oxygen permeable polyvinyl chloride film, and kept in darkness at 4 °C until being measured for color and heme pigment estimations. *LTL* color was measured as had been explained above, while heme pigments were measured as described in Lobón, Blanco, Sanz, Ripoll, Bertolín and Joy [4]. The 0-d samples were allowed to bloom also in darkness at 4 °C for 1 h before being measured. After that, meat was freeze-dried and vacuum-stored in total darkness at −80 °C until the analysis of polyphenols and a 2,2-azinobis-3-ethylbensothiazoline-6-sulfonic acid (ABTS) assay, which were determined according to Leal, et al. [20] and Vázquez, et al. [21], respectively.

### 2.5. Plasma Analysis

Plasma concentrations of creatinine and urea (kinetic methods) were analyzed with an automatic analyzer (GernonStar, RAL/TRANSASIA, Dabhel, India). The methodology to determine antioxidant activity of plasma regarding to polyphenols concentration, super-oxide dismutase (SOD) and ABTS and the method of the determination of lipid oxidation (measured as malondialdehyde; MDA) are described in Baila, et al. [17].

### 2.6. Statistical Analysis

Data were analysed with the SAS [22] using the lamb as the experimental unit. The productive traits—animal performances and plasmatic metabolites—, carcass characteristics and meat chemical composition of the suckling lambs were analyzed through an variance analysis with a general linear model, with the presence of PAC as the fixed effect. Color and heme pigments of *LTL* muscle were analysed with mixed models—MIXED procedure—with presence of PAC, time of display and their interactions as fixed effects and the lamb as the random effect. The degrees of freedom were adjusted with the Kenward-Rodger correction. Results were reported as least square means and their associated standard errors of the means, and the Tukey correction was applied for pair-wise comparisons. The effects were considered significant at *p* < 0.05.

## 3. Results

### 3.1. Lamb Performance and Plasma Metabolites

The productive traits of the suckling lambs are shown in Table 1. The presence of PAC in the dams’ diet did not affect any productive trait studied, such as average daily gain, age and weight at slaughter. Regarding the plasma metabolites at slaughter, the presence of PAC in the dams’ diet did not affect creatinine and urea plasmatic concentrations of the suckling lambs.

In the same line, the plasma polyphenols concentration and the antioxidant activity measured as SOD, ABTS and MDA were not affected by the presence of PAC in the dams’ diet (*p* > 0.05).

### 3.2. Digestive Compartments and Carcass Traits

The presence of PAC in the dams’ diet significantly increased the weight of the content in the reticulum-rumen (*p* < 0.05) and, concomitantly, in the forestomach (*p* < 0.01) and increased the weight of the digestive compartments (*p* < 0.05), except for the omasum-abomasum (Table 2). Nevertheless, most of the carcass characteristics were not affected by the treatment (Table 2). The color of subcutaneous fat of the suckling lambs was similar between groups, except for lightness, which was decreased with the presence of PAC in the dams’ diet (*p* < 0.01).

### 3.3. Meat Quality

There were no differences between groups in the pH values of *LTL* at 24 h *post-mortem*, DM, CP, intramuscular fat, total collagen and ash contents (Table 3). The polyphenol content of meat significantly decreased with the presence of PAC in the dams’ diet (*p* < 0.05) but did not affect the total antioxidant capacity estimated by ABTS content.

**Figure 1:**
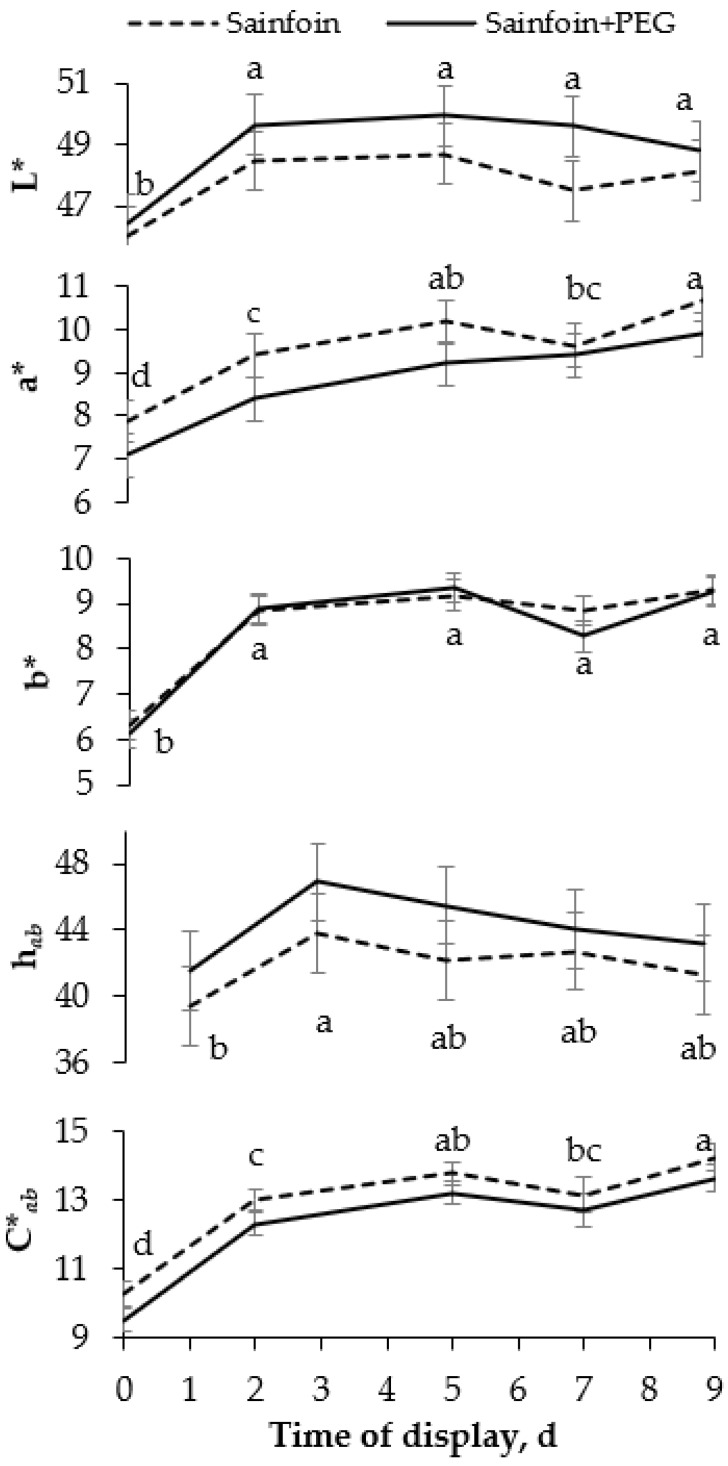
Evolution of instrumental color [lightness (L*), redness (a*), yellowness (b*), hue angle (h*_ab_*), and chroma (C*_ab_)] of meat of suckling lambs according to the presence of proanthocyanidins (PAC) in their dams’ diet. Within a parameter, different letters mean differences at *p* < 0.05 among days. Vertical bars indicate the standard error of the mean.

**Figure 2:**
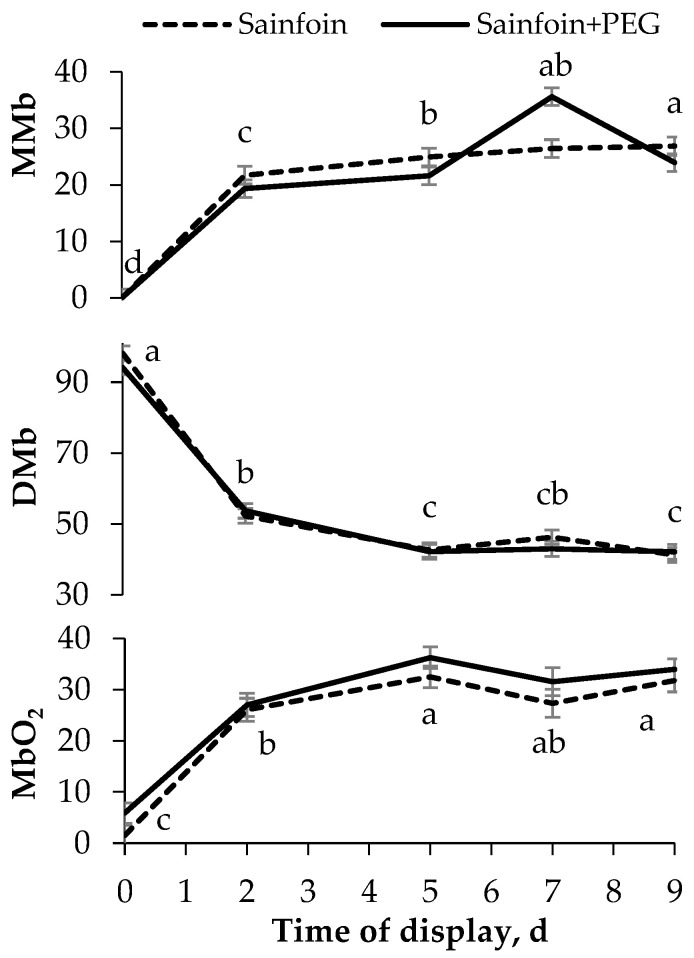
Evolution of heme pigments [metmyoglobin (MMb), deoxymyoglobin (DMb), and oxymyoglobin (MbO_2_)] of meat of suckling lambs according to the presence of proanthocyanidins (PAC) in their dams’ diet. Within a parameter, different letters mean differences at *p* < 0.05 among days. Vertical bars indicate the standard error of the mean.

No significant interactions were observed between the presence of PAC in the dams’ diet and the time of display of meat. The *LTL* color (Figure 1) was not modified by the presence of PAC on the dams’ diet, but it was affected by the time of display (*p* < 0.05). All color variables increased from day 0 to day 2 (*p* < 0.05), and thereafter L* and b* remained steady, whereas C*_ab_ and a* increased until day 5, remaining unchanged onwards (*p* < 0.001). Similarly to the color parameters, the presence of PAC in the dams’ diet had no significant effect on heme pigments (Figure 2), but the day of display had a significant effect (*p* < 0.05). Metmyoglobin and oxymyoglobin showed a similar evolution over time, increasing until day 5 (*p* < 0.001), whereas deoxymyoglobin followed the inverse pattern, decreasing until 5 day (*p* < 0.001) and remaining steady thereafter.

## 4. Discussion

The lack of effect of the presence of PAC from sainfoin in the diet of lactating ewes on the weight gains and carcass characteristics of their suckling lambs in the current study can be related to the similar milk production and quality of the dams, as previously shown. Milk yield and composition are the main factors responsible for suckling lamb growth [23], the protein intake being the most determinant [24]. A previous study [10] reported a higher ADG in suckling lambs whose dams received polyphenols of grape seed extract, but not when the supplementation was given directly to fattening lambs. Therefore, it is possible that polyphenols elicit a more pronounced effect during the suckling period compared to the post-weaning phase, as shown in lambs supplemented with grape pomace [25]. In a meta-analysis, it was reported that the performance of weaned lambs was not modified when the concentrations of PAC in their diet ranged between 16 and 25 g PAC/kg DM [26].

The growth of the suckling lambs of this study was comparable to that observed when dams were fed concentrates indoors, and higher than those obtained with dams fed on pasture during lactation [27]. Therefore, in this study, a diet based mainly on fresh sainfoin with only a 10% of supplementation was sufficient to achieve good performances in rearing a male lamb of this autochthonous breed. This alternative feeding management could be advisable to allow diversification of the production system and to increase system resilience in an unfavorable situation from a meteorological, social and economic point of view [28].

The similar plasma urea concentration at slaughter of lambs of both treatments was unexpected, because dams from the Sainfoin group had lower urea concentrations both in the plasma and the milk [17]. The Sainfoin + PEG suckling lambs ingested a greater quantity of urea from their dam’s milk, but it was not reflected in their plasma concentration, which suggests that the protein metabolism could be different between groups. The plasma creatinine concentration in suckling lambs was similar between treatments, indicating a similar metabolism of muscle mass [29], so that the PAC of dams’ diet had no effect on the use of amino acids of their lambs by reducing the ruminal degradation of dietary protein [16].

The antioxidant effect of PAC is well known [14,15], and some studies even report the transfer of dietary phenolic compounds from the milk to the meat of the suckling lamb, where they act as antioxidants [30]. However, in this study it was not observed in dams’ milk, which was reflected in the similar plasma antioxidant activity of lambs from both treatments. The metabolism of PAC along the digestive tract is complex [31], and the fact that the milk ingested by the lambs has already been processed in the mammary gland complicates the mechanism even further. In addition, Leparmarai, et al. [8] suggested that most of the phenolic compounds ingested by sheep were catabolized before reaching the milk or blood, so that the actual amount received by the lambs through the milk could be very low.

The carcass characteristics, dressing percentage and fat cover was similar in the suckling lambs of both experimental groups. Differences observed in carcass weight, fatness score and perirenal fat weight are usually related to the quality of feeding sources [32,33], dry matter intake [5,34,35], and/or age and weight at slaughter [33], all of which were similar in the present study. On average, the dressing percentage obtained in the suckling lambs was greater than the observed in fattened lambs, due to their lower development of the digestive tract of the former [5,33].

Unexpectedly, the reticulum-rumen and forestomach contents and the empty digestive compartments were heavier in lambs of the Sainfoin group. The higher weight of the digestive tract is usually associated with a worst dressing percentage [36]. In this case, the greater weight of the digestive organs of the Sainfoin group lambs produced a numerically lower dressing percentage, although the differences were not statistically significant. On the other hand, the digestive content is closely related with the intake. Since the milk intake of the lambs of both treatments was similar, the cause of this result remains unclear.

Yellowness and SUM (an estimator of carotenoids) in animal fat and meat are mainly influenced by the carotenoid pigments coming from feedstuffs [5,37]. The lack of differences observed in these parameters between treatments is ascribed to the similar sainfoin intake of their dams, and the consequent similar intake of secondary compounds. The lower L* value of caudal fat in Sainfoin lambs disagrees with the results observed by Rivaroli et al. [35], who concluded that the mechanisms of PAC modifying the L* of caudal fat deposits are unclear. Brainard [38] developed a method to estimate if instrumental color differences (expressed as $\Delta E_{ab}^{*}$) are perceptible by human vision. In relation to this, Carrasco, et al. [39] reported that differences of color ($\Delta E_{ab}^{*}$) of caudal fat lower than 5.2 between carcasses were imperceptible. In the present study, the difference between both treatments was 2.6; therefore, the practical implications were minimal. Fat L* values in suckling lambs of Churra Tensina, a similar local breed, ranged between 69 and 72 when their dams received a diet based on fresh forage or hay [40]. On the other hand, the color parameters were similar to those obtained by Lobón et al. [41] in fattened lambs previously raised by dams grazing sainfoin, despite their post-weaning finishing period and, consequently, heavier slaughter weight (23.4 kg BW).

Regarding the meat quality, the pH value of meat was within the normal range, close to 5.50 [27]. The presence of PAC in the dams’ diet did not have any effect on the chemical composition of the meat of their suckling lambs, as Gómez-Cortés et al. [42] reported when grape pomace was included as a source of PAC in the diet of lactating ewes. The content of fat and protein in the meat were similar to those obtained in suckling lambs of dams fed with pasture [27] or supplemented with polyphenols [10].

In this study, the content of polyphenol in the meat of suckling lambs reflects the concentration in the milk of their dams. Proanthocyanidins and polyphenols are characterized by providing a great antioxidant power [14] and studies show an improvement of antioxidant status which increases the shelf life of lamb meat [7,43]. However, this result was not reflected on the antioxidant capacity, probably because it was only measured on the first day post slaughter, when the oxidation process had hardly started.

In relation to heminic pigments in *LTL*, Vieira et al. [30] observed a reduction of MMb in the meat of suckling lambs whose dams were fed grape pomace (containing PAC). However, this reduction was observed from day 10 of meat storage, so the time of the present study could have been insufficient to show effects. Contrary to the present results of color, Vasta, et al. [44] in a review pointed out that the meat of lambs fed with PAC was lighter than that of their counterparts fed with PEG, concluding that the mechanisms of action of PAC on meat color are still unclear. Besides in the abovementioned review most of studies involved weaned lambs, whereas here we studied suckling lambs fed exclusively with maternal milk.

## 5. Conclusions

The inclusion of PAC from sainfoin in the dams’ diet had no significant effect on the ADG, plasmatic antioxidant activity, and carcass and meat quality of their suckling lambs. Therefore, fresh sainfoin can be fed to ewes during lactation to produce suckling lambs, achieving good performances and meat quality.

## Figures and Tables

**Table 1:** Effect of the presence of proanthocyanidins (PAC) in the dams’ diet ^1^ on the performance, plasma metabolites and antioxidant (AO) status of their suckling lambs.

Item	Sainfoin	Sainfoin + PEG	s.e.m ^2^	*p*-Value
Birth weight, kg	4.0	4.2	0.29	0.59
Average daily gain, g/d	272	283	21.0	0.63
Slaughter age, d	28.1	25.7	2.38	0.33
Slaughter weight, kg	11.6	11.1	0.33	0.14
Plasma metabolites				
Creatinine, µmol/L	52.2	55.4	7.28	0.67
Urea, mmol/L	4.86	5.16	0.619	0.63
Polyphenols, eq. [gallic acid] mg/mL	1.62	1.70	1.160	0.49
Antioxidant status				
Superoxide dismutase (SOD), U/mL	0.68	0.63	0.115	0.65
Total AO capacity–ABTS ^3^	5.32	5.79	0.294	0.13
Lipid oxidation, µΜ MDA ^4^	9.69	9.51	0.699	0.31

^1^ Sainfoin: lambs whose dams were fed ad libitum sainfoin + 200 g/d barley; Sainfoin + PEG: lambs whose ewes were fed ad libitum sainfoin + 200 g/d barley + 100 g/d polyethylene glycol (PEG); ^2^ standard error of the mean; ^3^ 2,2-azinobis-(3-ethylbensothiazoline)-6-sulfonic acid, µmol eq. [TROLOX]/mL; ^4^ malondialdehyde.

**Table 2:** Effect of the presence of proanthocyanidins (PAC) in the dams’ diet ^1^ on the weights of the digestive compartments, the carcass characteristics and the color of caudal fat deposits of their suckling lambs.

Item	Sainfoin	Sainfoin + PEG	s.e.m ^2^	*p*-Value
Weight of digestive content, g fresh matter (FM)				
Reticulum-rumen	284	192	39.5	0.033
Omasum-abomasum	185	161	41.6	0.57
Forestomach	469	353	47.5	0.011
Duodenum-jejunum	69	46	18.4	0.23
Weight of digestive compartments, g FM				
Reticulum-rumen	126	78	16.3	0.009
Omasum-abomasum	95	73	14.8	0.15
Forestomach	221	151	17.2	0.001
Duodenum-jejunum	171	132	13.8	0.011
Carcass traits				
Hot carcass weight, kg	7.78	7.64	0.264	0.61
Cold carcass weight, kg	6.19	6.15	0.238	0.87
Dressing percentage, %	55.6	57.2	1.03	0.13
Carcass shrinkage, %	3.98	2.99	0.595	0.12
Fatness score, 1–4 scale	2.10	2.15	0.103	0.74
Perirenal fat weight, g	128	129	24.7	0.98
Color of caudal fat deposits				
Lightness (L*)	68.8	71.4	0.48	0.015
Redness (a*)	2.6	2.5	0.31	0.89
Yellowness (b*)	12.3	11.9	0.45	0.66
Hue angle (h*_ab_*)	78.2	78.9	0.16	0.77
Chroma (C*_ab_)	12.6	12.2	0.49	0.71
SUM ^3^	102	110	13.1	0.75

^1^ Sainfoin: lambs whose dams were fed ad libitum sainfoin + 200 g/d barley; Sainfoin + PEG: lambs whose ewes were fed ad libitum sainfoin + 200 g/d barley + 100 g/d polyethylene glycol (PEG); ^2^ standard error of the mean; ^3^ estimator of carotenoids.

**Table 3:** Effect of the presence of proanthocyanidins (PAC) in the dams’ diet ^1^ on the pH, chemical composition and total antioxidant (AO) capacity of the meat of their suckling lambs.

Item	Sainfoin	Sainfoin + PEG	s.e.m ^2^	*p*-Value
pH _24 h_	5.5	5.53	0.032	0.46
Dry matter, % fresh matter (FM)	21.0	21.0	0.07	0.85
Crude protein, % FM	21.0	21.3	0.28	0.17
Intramuscular fat, % FM	2.36	2.19	0.124	0.18
Total collagen, % FM	0.77	0.77	0.153	0.99
Ash, % FM	1.81	1.78	0.103	0.75
Polyphenols, µg eq gallic acid/g FM	71.0	81.5	4.17	0.021
Total AO capacity-ABTS ^3^	0.44	0.45	0.035	0.76

^1^ Sainfoin: lambs whose dams were fed ad libitum sainfoin + 200 g/d barley; Sainfoin + PEG: lambs whose ewes were fed ad libitum sainfoin + 200 g/d barley + 100 g/d polyethylene glycol (PEG); ^2^ standard error of the mean; ^3^ 2,2-azinobis-(3-ethylbensothiazoline)-6-sulfonic acid, µmol eq. [TROLOX]/g FM.

## Data Availability

The datasets analyzed in the present study are available from the corresponding author on reasonable request.
